# Hereditary angioedema in women

**DOI:** 10.1186/1710-1492-6-17

**Published:** 2010-07-28

**Authors:** Laurence Bouillet

**Affiliations:** 1 National French Reference Centre of Angioedema, Internal Medicine Department, Grenoble University Hospital, France

## Abstract

Women with hereditary angioedema (HAE) are more likely to be symptomatic that men. Hormonal factors (puberty, contraception, pregnancy,....) play a significant role in the precipitation or worsening of the condition in women. So, combined contraceptive pills are not indicated and progestogen pill must be preferred. During pregnancy, attack rate can increase (38-48% of women). C1Inhibitor concentrate and tranexamic acid can be used during pregnancy. Attenuated androgens for long term prophylaxis are effective but side effects appear more often in female patients. These side effects are dose dependant and can be attenuated by titrating the dose down the lowest effective level.

## Review

Hereditary angioedema (HAE) is inherited in an autosomal dominant manner: consequently both women and men can be affected. However, published series of hereditary angioedema report a clear female predominance (60%) [1,2]. This might be explained by the fact that women are more likely to be symptomatic than men. In HAE associated with C1 Inh deficiency, Professor Bork has shown that women have more clinical episodes than men (p < 0.02) [2].

Hormonal factors play a significant role in the precipitation or worsening of the condition in women. There appear to be variation in overall frequency of angioedema symptoms according to the different female life stages of childhood, puberty, menses, pregnancies and menopause. Reports have noted a close relationship between female hormones and angioedema: a mother and her daughter whose HAE-related symptoms appeared to be sex hormone dependent [3]. Their first attack happened around puberty; angioedema worsened premenstrual and when they took combined oral contraceptives. The case of a woman [4] with HAE and Turner's syndrome is also very interesting: starting physiological oestrogen replacement at the age of 34 years old, this woman experienced a worsening both in the severity and in the frequency of angioedema attacks. McGlinchey and al [5] described a patient whose symptoms of HAE emerged after starting hormone replacement therapy (HRT).

Female sex hormones are known to affect the synthesis of many proteins. In the context of bradykinin mediated angioedema, they act on the kallikrein-kinin system by increasing synthesis of bradykinin. In ovariectomized rats, studies showed that 17β-estradiol increases Hageman factor levels by stimulation of gene transcription [6-9]. This hormone also increases kininogen and kallikrein levels [10]. Additionally oestrogens regulate B2 receptor gene expression and function: the vasodepressor response to bradykinin and the B2 receptor mRNA levels are reduced in ovariectomized rats, and restored by oestrogen substitution [11]. Progesterone does not modify Hageman factor levels but seems to raise kallikrein cDNA levels [12].

In healthy women taking oral contraception, there is an increase of fibrinolytic proteins: elevation of plasmin, factors VII, X, IX and a decrease of plasminogen activator inhibitor (PAI) [12-14]. These effects appear to be oestrogen-dependant [13]. The plasma of these women shows enhanced in vitro fibrinolysis [15]. The contact system is also affected: Hageman factor, prekallikrein, kallikrein and high molecular weight kininogen increase [16-19]. This results in consumption of C1Inh; the decrease of C1Inh levels correlating with the increase in Hageman factor [15,16]. Hormone replacement therapy (HRT) appears to have the same effect, despite lower oestrogen dose: fibrinolytic proteins (plasminogen and tissue-type plasminogen activator) rise, PAI decreases [19-21], Hageman factor, prekallikrein and C3, C4 levels rise [14,20,21]. Moreover, some studies have shown an influence of HRT on the bradykinin system: angiotensin converting enzyme activity decreases whereas bradykinin levels increase [22-24]. Visy and al [25] measured serum sex hormone levels in 44 females with HAE: they found a positive correlation between the rate of attacks and oestradiol and progesterone levels. However we don't have any information about clinical hormone sensibility women profile in this study.

It is generally accepted that there are distinct patterns of HAE in women. We delineate three of them below:

- Oestrogen dependent: these patients reveal the condition only when they are exposed to the combined contraceptive pill or during pregnancy. They usually have type III HAE.

- Oestrogen sensitive: the symptoms in these subjects are worsened by taking combined contraceptive medication or during pregnancy. Any type of HAE can present in this way.

- Oestrogen-independent: the use of the combined contraceptive pill or pregnancy does not exacerbate the symptoms. These individuals represent a minority of HAE patients.

The relationship between female hormones and angioedema appeared to be even clearer when the type III hereditary angioedema was recognised. This HAE mostly affects women. It was initially described by Bork et al, Binkley et al, and Martin et al in 2000 as recurrent angio-oedema without quantitative or functional C1Inh abnormalities [26-28]. In 2006, Dewald G (et al.) and Cichon (et al.) identified two mutations in the *F12 *gene (gene encoding for Hageman factor) associated with type III HAE [29,30]. Only 15-20% of the patients suffering from type III HAE had one of these mutations.

The clinical characteristics of type III HAE attacks are the same as for types I and II, although Bork suggested that facial swelling occurred considerably more often [31,32]. In terms of the effect of estrogens, although, AE attacks occurred preferentially in women taking the OC pill or during pregnancy [33,34]. Whilst the attacks appeared to be estrogen-dependent in Binkley's series (in which attacks began in the 15 days following starting oral contraception), they were only estrogen sensitive in the cases reported by Bork and Martin (estrogen exposure could induce attacks but after varying periods of time) [26-28]. We reported that 54.5% of women are estrogen sensitive and 23% are estrogen dependent, confirming the potential involvement of estrogen, although the time between estrogen exposure and onset of the disease could vary from a few months to eight years [35].

When a physician takes care of women with a HAE, some issues have to be addressed: the choice of contraception, management of pregnancies and deliveries and the selection of an effective prophylactic treatment without side effects.

### Contraception

Combined contraceptive pills exacerbate symptoms in 63-80% of women [3,36-38]. This method of contraception is, therefore, contra-indicated in women with hereditary angioedema. A progestogen pill **(**mini or full dose) should be advised in this situation. However, if a patient is not having problems with the combined pill, there is no need to stop it. An intra-uterine device is a good alternative method and is generally very well tolerated [36].

### Pregnancy

Fertility and the rate of spontaneous abortion are the same as those found in the normal population. In one third of cases, pregnancy worsens symptoms, but in another third the symptoms are improved [36]. Attack rates increase during pregnancy especially during the third trimester [39,40]. During pregnancy it is acceptable to continue background treatment with tranexamic acid [41]. Danazol is contra-indicated. Treatment of severe attacks is based on the use of C1Inh concentrate [40-42].

The management of labour depends on how the pregnancy has progressed. If the patient has suffered worsening of the condition with frequent severe episodes, then labour must be covered with C1 Inh concentrate (20U/kg by IV infusion). If the disease has been less severe, there is no need for prophylaxis with C1 Inh concentrate. However, this should be available in the delivery room in case it is required. Epidural analgesia is not only acceptable, but is strongly recommended. The Caesarean section rate is no higher in these patients than in the general population.

### Lactation

There is no problem with breast-feeding. However, tranexamic acid and danazol should not be taken as they are secreted in maternal milk. For the same reason icatibant should be avoided and only C1Inh concentrate should be used in the treatment of severe episodes [39].

### Menopause

In most patients (55%) the menopause does not alter the disease. One third is worse while only 13% improve [36]. Menopausal hormone replacement therapy should not be given because oestrogen can exacerbate the condition [5].

### Breast cancer

The incidence of breast cancer is no higher than in the rest of the population. Tamoxifen should not be used as it may worsen symptoms [43].

Women need also specific management for treatment of HAE.

Short term prophylaxis: three options are available: attenuated androgens, tranexamic acid or C1Inh concentrate. There is no specific problem for the use of theses drugs for short course in female patients. In case of short term prophylaxis with attenuated androgens, no virilisation has been observed [44,45].

Acute attack treatment: there is no specific problem for the use of C1inh concentrate, tranexamic acid, icatibant; or ecallantide in female patients.

### Long term prophylaxis

Antifibrinolytiques (acid tranexamic) are the first best choice for HAE women because of good tolerance. The limits are a moderate efficacy and adverse effects as nausea, diarrhea and theoretical risk about thromboembolism. These products present no specific effect for women. Only few women have reported mild dysmenorrhea [46,47].

Attenuated androgens are highly effective but are accompanied by side effects. These side effects appear more often in female patients. The result of PREHAET study (presented by Bork) reported a weight gain for 30% of women, virilisation for 6%, menstrual irregularities for 30%, acne for 7%. Women report also alopecia, hirsutism, and mammary hypotrophy [48-50]. The side effects are dose dependant and can be attenuated by titrating the dose down the lowest effective level [51-53]. It is important to note that women who take this treatment may ovulate even if they present menstrual irregularities or amenorrhea. So, it's important to use additional contraceptive method for fertile women taking attenuated androgens. This treatment must be stopped in case of pregnancy and lactation.

## Competing interests

The authors declare that they have no competing interests.
